# The effect of natural selection on the performance of maximum parsimony

**DOI:** 10.1186/1471-2148-7-94

**Published:** 2007-06-25

**Authors:** Dehua Hang, Eric Torng, Charles Ofria, Thomas M Schmidt

**Affiliations:** 1 Department of Computer Science and Engineering, Michigan State University, East Lansing, MI 48824, USA; 2 Department of Microbiology and Molecular Genetics, Michigan State University, East Lansing, MI 48824, USA

## Abstract

**Background:**

Maximum parsimony is one of the most commonly used and extensively studied phylogeny reconstruction methods. While current evaluation methodologies such as computer simulations provide insight into how well maximum parsimony reconstructs phylogenies, they tell us little about how well maximum parsimony performs on taxa drawn from populations of organisms that evolved subject to *natural selection *in addition to the random factors of drift and mutation. It is clear that natural selection has a significant impact on *Among Site Rate Variation *(ASRV) and the rate of accepted substitutions; that is, accepted mutations do not occur with uniform probability along the genome and some substitutions are more likely to occur than other substitutions. However, little is know about how ASRV and non-uniform character substitutions impact the performance of reconstruction methods such as maximum parsimony. To gain insight into these issues, we study how well maximum parsimony performs with data generated by Avida, a digital life platform where populations of digital organisms evolve subject to natural selective pressures.

**Results:**

We first identify conditions where natural selection does affect maximum parsimony's reconstruction accuracy. In general, as we increase the probability that a significant adaptation will occur in an intermediate ancestor, the performance of maximum parsimony improves. In fact, maximum parsimony can correctly reconstruct small 4 taxa trees on data that have received surprisingly many mutations if the intermediate ancestor has received a significant adaptation. We demonstrate that this improved performance of maximum parsimony is attributable more to ASRV than to non-uniform character substitutions.

**Conclusion:**

Maximum parsimony, as well as most other phylogeny reconstruction methods, may perform significantly better on actual biological data than is currently suggested by computer simulation studies because of natural selection. This is largely due to specific sites becoming fixed in the genome that perform functions associated with an improved fitness.

## Background

One of the most important problems in systematic biology is *phylogenetic tree reconstruction*. The use of phylogenetic trees is a fundamental step in many biological problems, such as the inference of evolutionary relationships among genes, genomes and organisms, protein structure and function prediction, and drug design [1].

Because of the importance of phylogeny reconstruction, many different reconstruction techniques have been developed. One of the most popular and widely used techniques is maximum parsimony [2]. The basic idea behind maximum parsimony is to find a most parsimonious phylogenetic tree; that is, a tree that requires the fewest mutations to explain the observed sequences. One drawback of maximum parsimony is its computational complexity. Finding a most parsimonious tree is an NP-hard problem [3], which means that it is unlikely any algorithm can find a most parsimonious tree quickly for all possible input sequences. One of the best implementations of maximum parsimony is that of [4] which can handle roughly 1000 taxa.

Evaluating how well maximum parsimony or any other technique reconstructs phylogenies is a difficult problem. Several different evaluation methodologies have been proposed [5] including computer simulation [6-9] and experiments with organisms with known phylogenies [10,11]. Unfortunately, each of these techniques is handicapped by at least one of the following drawbacks. The true phylogeny is not known. The simulated taxa have no meaning and do not contain genes. The simulated taxa have been generated by simplifying the evolutionary processes to ignore important mechanisms such as natural selection. There is limited data from which to draw statistically robust conclusions about the relative performance of algorithms. The result is that existing evaluation methodologies have left us incapable of answering several fundamental questions about phylogeny reconstruction. What is the effect of natural selection on the performance of a phylogeny reconstruction technique such as maximum parsimony? What is the effect of using the wrong character substitution model on the performance of a phylogeny reconstruction technique such as maximum parsimony? What is the effect of using the wrong mutation location model on the performance of a phylogeny reconstruction model such as maximum parsimony?

For example, consider the evaluation method of generating synthetic data using computer simulation techniques [6-9]. This method has many advantages including that the true phylogeny is known and large amounts of data can be generated rapidly. To generate synthetic data using a computer simulated evolutionary process, the researcher typically specifies four model components: a tree topology, a character substitution model, a mutation location model, and a probability distribution for the ancestral root sequence. A specific ancestral sequence is generated from the probability distribution, and the simulation mutates the sequence down the given tree topology according to the given substitution and mutation location models until a sequence has been generated for each leaf of the tree. Two programs commonly used to generate sequences according to a given tree and substitution model are Seq-Gen and PSeq-Gen [12,13]. The character substitution model is used to simulate varying probabilities of accepted mutations. In natural sequences, the probability of mutating from one character to another and having this mutation be accepted depends on the specific characters involved. Several different amino acid and nucleotide substitution models have been developed to partially account for the effects of biochemistry and natural selection [14-18]. The mutation location model is used to simulate *Among Site Rate Variation *(ASRV). In natural sequences, different sites undergo substitutions at different rates; that is, the mutation location model is not uniform. For example, portions of the genome that correspond to binding or catalytic sites of protein sequences will experience fewer mutations than the non-coding portions of the genome. ASRV affects estimation of distances between sequences, transition rate bias and phylogeny reconstruction. Several different ASRV models have been proposed [19,20]. Usually a mathematical distribution, such as a gamma distribution, is used to model ASRV.

The key drawback of computer simulations is that despite the use of sophisticated models, the taxa are not collected from a natural evolutionary process. Instead, the taxa are simply random sequences of characters that have no meaning and do not include actual genes or functional components. Thus, no sequence can be "more fit" than another sequence, and the taxa are determined solely by mutation and drift but not natural selection. Therefore, computer simulation techniques cannot be used to assess the effect of natural selection on the performance of maximum parsimony.

Working with natural data with known phylogenies partially overcomes the limitations of computer simulation [10,11]. However, even these data suffer from important limitations. First and foremost, the number of known phylogenies is relatively small. Furthermore, in experimental phylogenies, the mutation rates typically are manipulated to increase divergence with the potential side effect of overemphasizing genetic drift as compared to natural selection. Finally, while the major phylogenetic relationships will be known, there is a limit to the amount of data available to the researcher. In computer simulations, for example, all mutation events can be recorded at a level of detail impossible with real biological organisms. Thus, we are still incapable of answering many questions about the effect of natural selection on reconstruction accuracy.

In this work, we study exactly this question. What is the effect of natural selection on the performance of a reconstruction technique such as maximum parsimony? Does it improve or hinder reconstruction accuracy? Furthermore, assuming that natural selection does influence reconstruction accuracy, how does it affect reconstruction accuracy? More specifically, we know that natural selection creates both ASRV and non-uniform character substitution. What is the relative importance of ASRV and non-uniform character substitution on reconstruction accuracy?

### The Avida digital evolution research platform

We study these questions using the Avida digital evolution research platform [21] to generate a collection of sequences that experience true Darwinian evolution from which we can attempt to reconstruct a phylogeny. In Avida, each sequence represents a self-replicating computer program. Like computer simulations, all information including the phylogeny of the sequences can be perfectly recorded. However, the Avida populations **do not simulate **evolution; they **evolve **subject to mutation, drift, and selection. We describe below how mutation and selection arise.

When a digital organism replicates, it copies its genome, one instruction at a time, into empty memory. Mutations arise due to a faulty copy command. When an instruction is copied, there is a small probability that it will be copied incorrectly. This probability or mutation rate is an experimental parameter set by the researcher. Typically, when a mutation occurs, an incorrect instruction is chosen uniformly at random from the remaining instructions, though we do consider other probability distributions as well.

Space is limited in an Avida population and death occurs when organisms are removed (at random) to make room for new offspring. The fitness of an organism is essentially its replication speed. One way for an organism to increase its fitness is for it to replicate using fewer instructions. An alternative is to perform specific Boolean logic tasks (akin to metabolic reactions) that are specified in the researcher-defined digital environment that give the organism extra energy that allows the organism to execute instructions more rapidly. For example, if NOR is a rewarded task, organisms can obtain extra energy by reading in two vectors of binary digits, performing NOR on each corresponding pair of bits in the two inputs, and outputting the resulting bit vector. There are many different ways to perform NOR and rarely, if ever, will evolution find identical solutions in replicate runs.

### The Avida system and natural selection

A concern that we are faced with in using Avida as a data source for phylogeny reconstruction studies is the same credibility gap that computer simulation studies face. Specifically, how similar is data from Avida experiments to data found in the natural world?

We first emphasize that Avida is a widely used platform for studying evolution. Several dozen peer reviewed papers have been published based on Avida experimental data including several in Nature, Science, and the Proceedings of the National Academy of Sciences [22-26].

Biological communities have accepted Avida experimental data because populations of digital organisms **undergo true Darwinian evolution **where individuals have genomes that are expressed into phenotypes. Avida experiments **do not simulate **any specific biological process or system. Instead, Avida organisms are computer programs with actual meanings and fitness values. Thus, Avida organisms **do evolve **subject to **natural selection**.

For example, Avida organisms are highly contingent on previous events. Ofria and others have shown that complex traits can evolve in Avida [25] if simpler traits are rewarded by using the genetic code that performs the simpler traits as building blocks. On the other hand, sometimes the use of a particular gene in one way will constrain further evolution in other directions. A specific case of this is that the replication of an organism's genome can become over-optimized relying on that genome to be a specific length, thereby constraining all other genes to exist within a fixed number of instructions and thus limiting their evolution. Wagenaar and Adami showed that chance events are required to open gateways that allow further adaptation [27]. Thus, Avida experiments show wide variation in the paths taken by evolution with replicate runs rarely finding the same solution. Epistasis also occurs in Avida [22] and the dynamics were essentially identical to those found in long term E. coli experiments [28].

It is true that Avida data does have some limitations. The genome length of an Avida organism in our experiments is 100 (with 26 possible instructions at each position). This implies that the genome of a typical Avida organism is less complex than that of most biological organisms, but is certainly comparable to that of short genes. Thus, Avida phylogenies can most easily be compared to gene phylogenies of biological organisms. While we could increase the length of the Avida organisms, it is not clear this increase would be meaningful. The information density of the longer organisms would be less than that of the length 100 organisms if both organisms perform the same set of tasks.

Another potential limitation is that Avida's basic life chemistry is distinct from that of natural organisms. However, while the specific chemistry may be different, we can still compare phylogeny reconstruction with and without natural selection to derive general principles about natural selection's effect on phylogeny reconstruction that should apply to any evolving system. Any such general principles should be robust across most biological systems.

We have previously used Avida to analyze the effect of natural selection on the performance of maximum parsimony and neighbor joining [29,30]. In [30], we demonstrated that maximum parsimony and neighbor joining could reconstruct 4 taxa phylogenies surprisingly well when all branches are extremely long and no significant functionality is being acquired through evolution. In [29], we tested a wider range of branch lengths for 4 taxa tree topologies and showed that both maximum parsimony and neighbor joining can reconstruct data sets generated with natural selection more accurately than datasets generated without natural selection, particularly when the internal branch is long enough to allow the acquisition of a significant phenotypic trait. We also showed that incorporating a simplistic ASRV model significantly enhanced the quality of the computer simulation data.

In this paper, we perform a more detailed analysis to determine how natural selection impacts the performance of maximum parsimony. We isolate specific conditions where the presence of natural selection significantly improves the reconstruction accuracy of maximum parsimony. We then illustrate how selective pressures cause changes in both ASRV and instruction frequency (which is related to the character substitution model). Our results show that ASRV and non-uniform character substitutions are not sufficient to capture the effects of natural selection on the performance of maximum parsimony. However, the computer simulation data produced by accurately simulating ASRV is more similar to real, experimental data than the computer simulation data produced by accurately simulating character substitution probabilities. Finally, we corroborate these results using PSeq-Gen data.

## Results and Discussion

**Figure 1:**
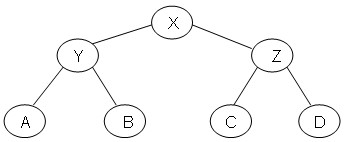
Model tree topology.

Our experimental methodology consists of two components that are described more completely in our methods section. In brief, we generate symmetric 4 taxa topologies to be reconstructed as depicted in Figure 1. The internal branch length of this 4 taxa topology is the number of unique genotypes in the phylogeny from the intermediate ancestor Y to the intermediate ancestor Z, one of which is the ultimate ancestor X. The external branch length is the number of unique genotypes from either intermediate ancestor Y or Z to any of the 4 descendant children A, B, C, or D.

We generate sequences that evolve subject to natural selection using Avida. We generate sequences that evolve without natural selection by using computer simulation techniques. Within Avida, we utilize different reward structures to vary the selective pressure. In a given reward structure, some set of tasks is rewarded along a given branch in the tree topology. The set of tasks rewarded might change from one branch to another.

### Conditions where natural selection improves reconstruction accuracy

**Figure 2:**
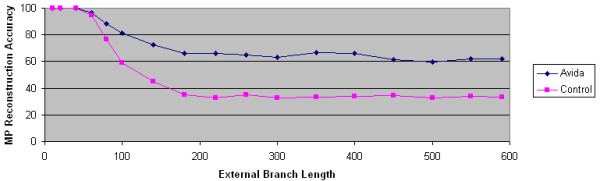
**Natural selection improves maximum parsimony's reconstruction accuracy**. The accuracy of maximum parsimony is plotted as a function of external branch length. The Avida data set is generated using the overlapping reward structure. The control data set is generated using computer simulation techniques. In both data sets, the internal branch length is 60.

We first determine if natural selection has any impact on the accuracy of maximum parsimony. Our initial results show that there are conditions where natural selection can significantly improve the accuracy of maximum parsimony, corroborating results from our earlier papers [29,30]. For example, consider the data from Figure 2. MP's reconstruction accuracy is significantly higher given the Avida data rather than the control data from computer simulation. In fact, MP can reconstruct 4 taxa trees with surprisingly large branch lengths.

**Figure 3:**
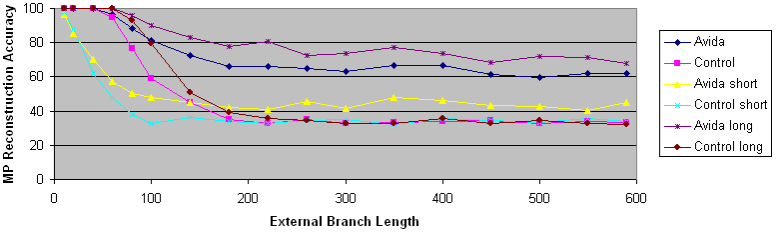
**The impact of internal branch length**. The accuracy of maximum parsimony is plotted as a function of external branch length. The Avida data sets are generated using the overlapping reward structure. The control data sets are generated using computer simulation techniques. In the short data sets, the internal branch length is 6. In the long data sets, the internal branch length is 200. In the default data sets, the internal branch length is 60.

We now isolate what conditions lead to natural selection improving MP's reconstruction accuracy. Our first test is to vary the internal branch length of the tree. Our results shown in Figure 3 demonstrate that natural selection's impact increases as we increase the internal branch length. For example, when the internal branch length is only 6, there is little difference between MP's accuracy on the two data sets. When the internal branch length increases to 200, MP's superiority on Avida data is even larger than when the internal branch length is 60. The explanation is that there must be enough time for significant adaptation to occur.

**Figure 4:**
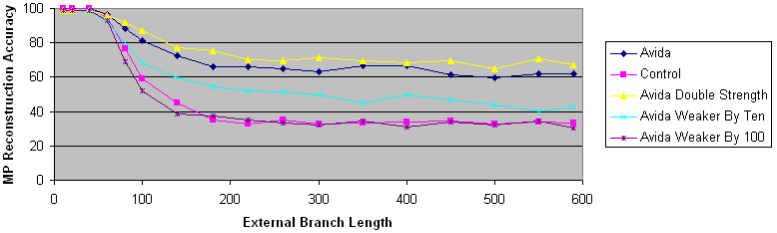
**The impact of selection strength**. The accuracy of maximum parsimony is plotted as a function of external branch length. The Avida data sets are generated using the overlapping reward structure. The control data set is generated using computer simulation techniques. In all data sets, the internal branch length is 60. The selection strength of the Avida data sets are given as a function of the default selection strength. For example, the selection strength in the "Avida weaker by 100" dataset is 100 times smaller than the selection strength in the default Avida data set.

We next experiment with selection strength to determine its impact on MP's reconstruction accuracy. Our results shown in Figure 4 demonstrate that natural selection's impact increases as we increase selection strength. For example, MP's reconstruction accuracy on data generated with a bonus for performing rewarded tasks that is 1/100^th ^of the default bonus is essentially identical to MP's accuracy on computer simulation data. As we increase the bonus, MP's accuracy improves.

**Figure 5:**
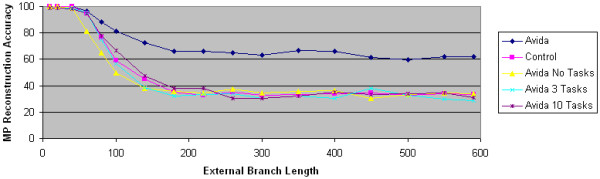
**The impact of changing reward structure**. The accuracy of maximum parsimony is plotted as a function of external branch length. The control data set is generated using computer simulation techniques. In all data sets, the internal branch length is 60. The Avida data set uses the overlapping reward structure. The other data sets reward 0, 3 or 10 tasks throughout the entire experiment.

We next experiment by varying the reward structure. In our default overlapping reward structure, new tasks are rewarded in each branch of the tree topology. We test the importance of changing selective pressures on MP's performance by assessing MP's reconstruction accuracy on Avida data generated where the same tasks are rewarded on all branches of the tree topology. Our results shown in Figure 5 demonstrate that changing selective pressures is critical; in particular, it is important that the intermediate ancestor incorporates a significant adaptation. When the same tasks are rewarded in all branches, most of the tasks are acquired by the original ancestor and thus no significant adaptations are incorporated into the intermediate ancestors.

**Figure 6:**
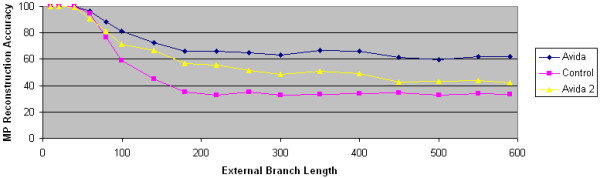
**Comparing overlapping and non-overlapping reward structures**. The accuracy of maximum parsimony is plotted as a function of external branch length. The Avida data set is generated using the overlapping reward structure. The Avida 2 data set is generated using the non-overlapping reward structure. The control data set is generated using computer simulation techniques. In all data sets, the internal branch length is 60.

We finally experiment by comparing the overlapping reward structure where some tasks are rewarded in consecutive branches in the tree topology with the non-overlapping reward structure where no task is rewarded in consecutive branches in the tree topology. Our results shown in Figure 6 demonstrate that MP's performance on both Avida data sets is significantly better than MP's performance on the control data generated using computer simulation. It is interesting to note that MP performs better on the Avida overlapping data set than the Avida non-overlapping data set. We believe this improved performance is due to the selective pressure to maintain critical pieces of code that are rewarded in consecutive environments (E1 and E2) as well as (E2 and E3). That is, a significant adaptation that is acquired in an intermediate ancestor may be lost if the associated task is no longer rewarded in the final environment E3. This is less likely to occur in the overlapping reward structure than the non-overlapping reward structure.

### The effect of selective pressure on among site rate variation (ASRV) and instruction frequency

Our results clearly demonstrate that there are situations where natural selection improves MP's reconstruction accuracy. To help determine which factor, ASRV or non-uniform accepted substitution probabilities, is the more influential byproduct of natural selection on the performance of maximum parsimony, we plot the ASRV and instruction frequencies that result from a variety of selective pressures. We use instruction frequency as a proxy for accepted substitution percentage because instruction frequencies are easier to interpret given the 26 instructions in the Avida instruction set. If we were to compare substitution percentages directly, that would require comparing 26^2 ^= 676 numbers while comparing instruction frequencies requires only 26 comparisons. Our results indicate a stronger relationship between ASRV and reward structure than between instruction frequencies and reward structures. This implies that ASRV may be a more important factor than non-uniform character substitution. However, our results also indicate that ASRV and non-uniform character substitutions cannot account for the entire effect of natural selection on the performance of maximum parsimony.

**Figure 7:**
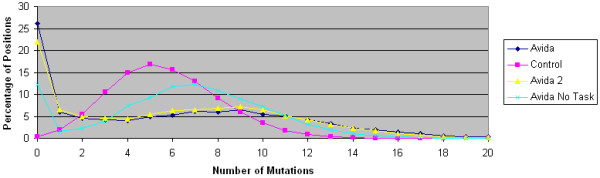
**Distribution of per site number of mutations**. The percentage of the 40,000 genomic positions or sites that experience a given number of mutations is plotted for the range 0 mutations through 20 mutations. The data was generated using 100 replicate experiments for each reward structure. Each replicate contributed 400 genomic positions or sites and used an internal branch length of 60 and an external branch length of 600.

To assess the relationship between ASRV and natural selection, we compare how the mutations are distributed by genomic position for 4 different selective pressures: Avida with the overlapping reward structure, Avida with the non-overlapping reward structure, Avida with the no-task reward structure, and the control data set generated by computer simulation. For each selective pressure, we collect data from our experiments with internal branch length 60 and external branch length 600; we have 100 replicates for each selective pressure. For each data set, we compute the number of mutations that occur in each of the 100 positions from an intermediate ancestor (Y or Z in Figure 1) to a leaf taxa (A, B, C or D in Figure 1). Since we have a total of 100 replicates with 4 lines of descent for each replicate and 100 genomic positions for each line of descent, there are a total of 40,000 unique positions. Due to the external branch length of 600, the average number of mutations that each position will experience will be approximately 6; it is slightly higher because there is a small chance that more than one mutation may occur between a parent and a child. The mutation distribution data for our 4 data sets is depicted in Figure 7.

We see that for the control data set with no selective pressure, the plot of the number of positions that experience a given number of mutations is, as expected, a normal distribution centered close to 6. Likewise for the no-task reward structure data set, once the positions with 0 mutations are deleted, the plot of the number of positions that experience a given number of mutations is also normally distributed with the center close to 7. This occurs because the main selective pressure in the no-task reward structure setting is to maintain the replication cycle. The instructions responsible for replication are the ones in positions with 0 mutations. On the other hand, in the non-overlapping and overlapping structure data sets, the plot of the number of positions that experience a given number of mutations is not a normal distribution but rather is close to uniform in the range from 1 mutation to 12 mutations with a significant tail for higher numbers of mutations. Thus, significant changes in ASRV do correlate with significant changes in MP's reconstruction accuracy. However, there is not a sharp difference in the mutation distribution plots for the overlapping and non-overlapping data sets. This suggests that ASRV alone is not sufficient to explain the improved performance of maximum parsimony on the data generated in the overlapping reward structure compared to the data generated in the non-overlapping reward structure.

**Figure 8:**
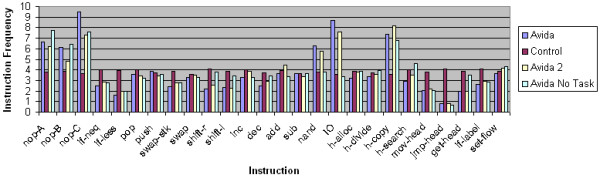
**Instruction frequencies**. For each data set; Avida with overlapping reward structure, Avida with non-overlapping reward structure, Avida with no-task reward structure, and control data generated by computer simulation; the frequency of each of the 26 unique instructions in the four leaf taxa is plotted. The data was generated using 100 replicate experiments for each data set with 400 instructions per replicate giving a total of 40,000 instructions. Each replicate used an internal branch length of 60 and an external branch length of 600.

To assess the relationship between instruction frequency and selective pressure, we compare the instruction frequencies of the terminal taxa for the same set of 4 selective pressures. We use the same settings we did for the ASRV data: internal branch length 60 and external branch length 600. Similar to the ASRV data set, we have a total of 40,000 instructions in the 4 terminal taxa for each of the 100 replicates. Given that the instruction set size is 26, the average instruction frequency should be 3.86%. The instruction frequencies for the 4 data sets are plotted in Figure 8.

We see that for the control data set with no selective pressure, the instruction frequency distribution is essentially uniform at 3.86%. However, the instruction frequencies for the three Avida data sets that experience natural selection are clearly not uniform. The frequencies for instructions critical to replication such as h-copy, nop-A, nop-B, and nop-C are significantly above 3.86% in all three Avida data sets. The instruction frequencies for nand and IO, both of which are critical for performing Boolean logic tasks, are also significantly above 3.86% in the overlapping and non-overlapping reward structure data sets where there is a selective pressure to perform Boolean logic tasks.

When comparing the instruction frequencies, we see very little difference between the two from the overlapping and non-overlapping reward structures despite the significant difference in MP's reconstruction accuracy. Furthermore, there is a significant difference between the no-task reward structure instruction frequencies and the control data instruction frequencies while MP reconstructs both data sets with essentially the same accuracy. This data suggests that while non-uniform instruction frequencies and non-uniform character substitutions are a significant result of natural selection, they may not explain why natural selection improves the performance of phylogeny reconstruction algorithms such as maximum parsimony in some cases.

### The effect of among site rate variation (ASRV) and non-uniform character substitutions on the performance of maximum parsimony

The previous data implied that ASRV is more likely to have an effect on maximum parsimony's performance than non-uniform character substitutions. In order to study this question more carefully, we augment our computer simulation program in order to perfectly mimic either the *location *of mutations that occurred in an Avida experiment or the *substitutions *that occurred in an Avida experiment. When the computer simulation program mimics the locations of mutations, we define this as *location simulation*. When the computer simulation model mimics the substitutions that occur, we define this as *substitution simulation*. When using location simulation, the character substitutions are chosen at random. When using substitution simulation, the mutation location is chosen uniformly at random among available locations. Note that in normal computer simulation, both mutation location and character substitution are chosen uniformly at random. We describe this in more detail in our methods section.

**Figure 9:**
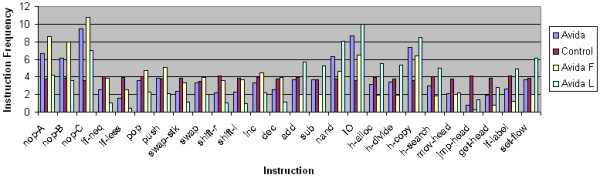
**Instruction frequencies with F and L substitution processes**. For Avida with the overlapping reward structure, the frequency of each of the 26 unique instructions in the four leaf taxa is plotted when the taxa are generated using the default uniform character substitution process, the F character substitution process and the L character substitution process. The data was generated using 100 replicate experiments with 400 instructions per replicate giving a total of 40,000 instructions. Each replicate used an internal branch length of 60 and an external branch length of 600.

Furthermore, in order to exaggerate the influence of the character substitution process, we augment the default Avida character substitution process that is uniform with two new character substitution processes, F and L, in which some substitutions are more likely to *occur *than others. Note this effect is distinct from that of natural selection in which case some mutations are more likely to be *accepted *than others. We provide more details on the F and L character substitution processes in our methods. As the data depicted in Figure 9 shows, using these biased character substitution processes does lead to instruction frequencies that are more divergent from uniform.

**Figure 10:**
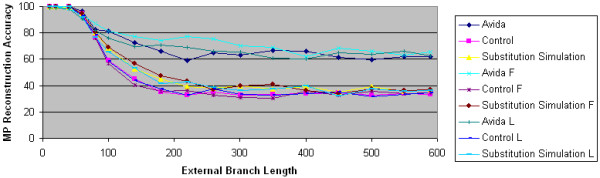
**Substitution simulation and maximum parsimony reconstruction accuracy**. The accuracy of maximum parsimony is plotted as a function of external branch length. The Avida data sets are generated using the overlapping reward structure with the default uniform, F and L character substitution processes. For each Avida data set, a control data set is generated using computer simulation techniques. Finally, for each Avida data set, a second control data set is generated using the substitution simulation technique. In all data sets, the internal branch length is 60, and there were 100 replicate experiments.

As Figure 10 shows, MP's performance on the substitution simulation data is essentially identical to its performance on the control data and is much worse than its performance on the Avida data. This is true even when we use the biased F and L character substitution processes. These results suggest that even though natural selection has a significant impact on character substitution probabilities, non-uniform character substitutions do not explain how natural selection improves maximum parsimony's improved reconstruction accuracy in some cases.

**Figure 11:**
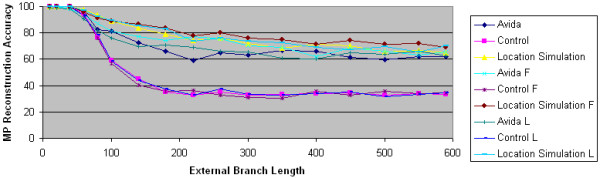
**Location simulation and maximum parsimony reconstruction accuracy**. The accuracy of maximum parsimony is plotted as a function of external branch length. The Avida data sets are generated using the overlapping reward structure with the default uniform, F and L character substitution processes. For each Avida data set, a control data set is generated using computer simulation techniques. Finally, for each Avida data set, a second control data set is generated using the location simulation technique. In all data sets, the internal branch length is 60, and there were 100 replicate experiments.

As Figure 11 shows, MP's performance on the location simulation data is very similar to its performance on the Avida data, even when the Avida data is generated using the biased F and L character substitution processes. These results suggest that one reason natural selection improves the performance of maximum parsimony in some cases is its introduction of ASRV.

An observation that is perhaps surprising is that MP actually performs better on the location simulation data than on the Avida data. This can be explained by the fact that homoplasy is less likely with a uniform character substitution model than a non-uniform character substitution model. Thus, in the genomic positions that experience high rates of change, more homoplasies are likely to occur in the actual Avida sequences than in the location simulation model sequences.

To further test the impact of ASRV and character substitution models on the performance of maximum parsimony, we performed comparable experiments using data from PSeq-Gen. For each of the four character substitution models (uniform, JTT, PAM, and mtREV), we generated 1000 replicates using both α = 1, which corresponds to significant ASRV, and α = 100, which corresponds to essentially no ASRV. For each data set, we computed MP's performance.

**Figure 12:**
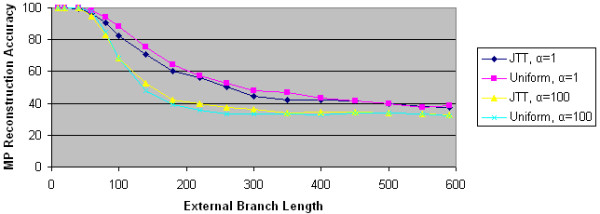
**MP reconstruction accuracies for PSeq-Gen JTT data**. We plot the maximum parsimony reconstruction accuracy as a function of external branch length for 4 PSeq-Gen generated data sets. Two data sets use the JTT character substitution model; the other two use the uniform character substitution model. Two data sets use α = 1 to model significant ASRV. Two data sets use α = 100 to model little ASRV.

**Figure 13:**
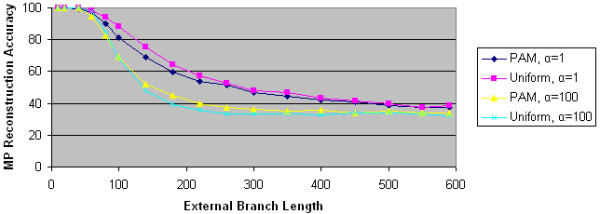
**MP reconstruction accuracies for PSeq-Gen PAM data**. We plot the maximum parsimony reconstruction accuracy as a function of external branch length for 4 PSeq-Gen generated data sets. Two data sets use the PAM character substitution model; the other two use the uniform character substitution model. Two data sets use α = 1 to model significant ASRV. Two data sets use α = 100 to model little ASRV.

**Figure 14:**
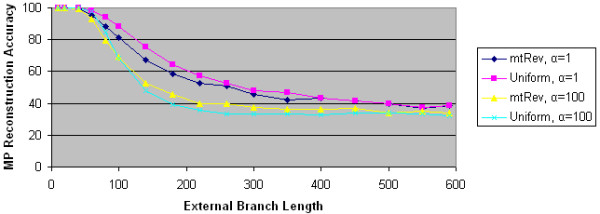
**MP reconstruction accuracies for PSeq-Gen mtREV data**. We plot the maximum parsimony reconstruction accuracy as a function of external branch length for 4 PSeq-Gen generated data sets. Two data sets use the mtREV character substitution model; the other two use the uniform character substitution model. Two data sets use α = 1 to model significant ASRV. Two data sets use α = 100 to model little ASRV.

As Figures 12, 13, and 14 show, none of the three character substitution models implemented in PSeq-Gen (JTT, PAM, and mtREV) has a significant effect on MP's reconstruction accuracy. More specifically, for either α value, MP's reconstruction accuracy is essentially identical using all four substitution models, including uniform. On the other hand, the α value and thus ASRV has a significant effect on MP's reconstruction accuracy regardless of the substitution model used. These results validate our findings with Avida data.

## Conclusion

Maximum parsimony may perform significantly better on actual biological data than is currently suggested by computer simulation studies due to the effects of natural selection. In sequences that evolve subject to natural selection and which experience a geographic separation, specific sites in the genome that perform critical functions become resistant to accepted mutations. As there are typically many distinct ways to perform a function, the solution derived by one population is likely to be different than the solution derived by the second population. The sites associated with these different solutions provide a signal that aids in the reconstruction of phylogenies. As this phenomenon is more strongly associated with locations rather than the specific substitutions that occur, ASRV appears to have a stronger impact on phylogeny reconstruction than character substitution probabilities.

It is important to note, however, that natural selection led to improved reconstruction accuracy for Maximum Parsimony only when there were changes in rewarded tasks that led to intermediate ancestors acquiring significant adaptations. This would suggest that natural selection will only help in reconstructing phylogenies from taxa that diverged due to new selective pressures. The prevalence of this phenomenon in the natural history of our world will determine to some extent the significance of this finding.

It is also important to note that we performed all our experiments using symmetric tree topologies. Maximum parsimony is known to perform poorly when the topologies are not symmetric [31-33]. It is a natural question to ask how the effects of natural selection interact with the issue of long branch attraction.

In future work, we plan to test whether our results and conclusions still hold when applied to larger phylogenies, to smaller character sets, or when our techniques are applied to tree reconstruction algorithms other than Maximum Parsimony. Furthermore, we plan to evaluate how well Maximum Parsimony performs on asymmetric tree topologies.

## Methods

Our basic methodology is simple. We first generate a set of four taxa with a known phylogeny that evolves subject to natural selection using Avida. We label the four taxa as A, B, C, and D and the three internal ancestors at bifurcation points as X, Y, and Z. See figure 1 for an illustration. Next, we generate three sets of computer simulation data based on our known phylogeny. We work with 4 taxa trees because we can compute the most parsimonious tree quickly and we can simplify the problem of determining if a tree is correct. Specifically, there are 3 distinct topologies that can be produced by a reconstruction algorithm; the correct one, which pairs A with B and C with D, or two possible false answers that incorrectly pair A with C and B with D, or incorrectly pair A with D and B with C.

We assess the performance of maximum parsimony for a given data set as follows. If maximum parsimony outputs only the correct tree topology, it is given a score of 1 since the correct answer is given unambiguously. If maximum parsimony outputs two possible tree topologies, one correct and one incorrect, it is given a score of 1/2 since the correct answer is given but an incorrect answer is given as well. If maximum parsimony outputs all three possible tree topologies, it is given a score of 1/3. If maximum parsimony fails to output the correct tree topology, it is given a score of 0. Note that randomly selecting a topology (or a combination of topologies) would have an expected accuracy of 1/3.

### Generation of known phylogenies with Avida

**Figure 15:**
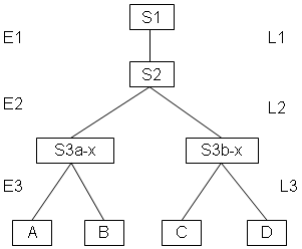
**Experimental procedure to generate Avida data**. A population seeded with organism S1 is allowed to reproduce in environment E1 for L1 generations. The dominant organism at the end of the experiment, S2, is then used to seed two new experiments in environment E2 for L2 generations. The dominant organisms from these two experiments, S3a-x and S3b-x, are each used to seed two new experiments in environment E3 for L3 generations. The dominant organisms from each of these 4 experiments, A, B, C, and D, represent a set of taxa to be reconstructed. The definition of generation with respect to L1, L2, and L3 requires that there be at least one mutation from parent to child.

We generated Avida data in the following manner, which is based on the approach taken by Hillis et al. with viruses [10,11]. The procedure is illustrated in Figure 15. First, we took a viable ancestor S1 and injected it into an initial environment E1. The ancestor was handwritten and contains a short copy loop and genome that is padded out to length 100 with inert no-operation instructions. During the Avida run, insertion or deletion mutations were disallowed. This ensured all genomes have length 100 and are aligned. Although the specific length 100 is somewhat arbitrary, it is enough to provide space for mutations and significant adaptation. All environments were limited to a population size of 3600. Previous work with Avida [22] has shown that 3600 is large enough to allow for diversity while making large experiments with many replicates practical.

We set the copy command to fail with a uniform probability of 0.75%, which means that for a typical replication, .75 (out of the 100) instructions would be copied incorrectly. When a copy command fails, a random instruction is written in its place, each with equal probability in our default setting. For some Avida experiments, we used two non-uniform character substitution processes F and L, which emphasize the first 13 instructions and the last 13 instructions, respectively. When the copy command fails with the F (L) character substitution process, each of the first (last) 13 instructions is written in its place with probability 4/65 and each of the last (first) 13 instructions is written in its place with probability 1/65.

After running for L1 = 200 generations, we identified the most abundant genotype S2 (or X from Figure 1) and placed S2 into a new environment E2. We executed two parallel experiments of S2 in E2 for 1.08 × 10^10 ^cycles, which is approximately 10^4 ^generations. In each of the two experiments, we then sampled genotypes at L2 equal to 3, 30, and 100 along the line of descent from S2 to the most abundant genotype at the end of the execution. Let S3a-x denote the sampled descendant in the first experiment for L2 = x while S3b-x denotes the same descendant in the second experiment. Note that S3a-x and S3b-x correspond to internal ancestors Y and Z from Figure 1.

Next, for each value x of L2, we took S3a-x and S3b-x and put them each into a new environment E3. Again, we executed two parallel experiments for each organism. In each of the four experiments, we then sampled genotypes at L3 equal to a set of values from 10 through 600 along the line of descent from S3a-x or S3b-x to the most abundant genotype at the end of the execution. For each value of L3, four taxa A, B, C and D were used for reconstruction. Organisms A and B share the same ancestor S3a-x while organisms C and D share the same ancestor S3b-x.

Within the environments E1, E2, and E3, there were energy bonuses associated with ten different Boolean logic tasks which, in order of complexity, are ECHO, NOT, NAND, AND, ORNOT, OR, ANDNOT, NOR, XOR, and EQUAL. We use many different reward structures for our Avida experiments. Our default reward structure is the overlapping reward structure where tasks ECHO, NOT, NAND, AND, and ORNOT are rewarded in E1, tasks AND, ORNOT, OR, and ANDNOT are rewarded in E2, and tasks ANDNOT, NOR, XOR, and EQUAL are rewarded in E3; note that there are tasks shared between E1 and E2 and between E2 and E3. We also consider the no-task reward structure where none of the ten logical tasks are rewarded in environments E1, E2 and E3; the non-overlapping reward structure where tasks ECHO, NOT, and NAND are rewarded in E1, tasks AND, ORNOT, and OR are rewarded in E2, and tasks ANDNOT, NOR, XOR, and EQUAL are rewarded in E3; the 3-tasks reward structure where tasks ECHO, NOT, and NAND are rewarded in environments E1, E2 and E3; and the 10-tasks reward structure where all 10 Boolean logic tasks are rewarded in environments E1, E2, and E3.

The internal branch length of our tree structure is twice the value of L2, for which we used values 3, 30 and 100. Note that L2 = 3 means that, for example, genotype S2 is genotype S3a-3's great grandparent. Typically, the number of mutations that differentiate a parent from a child is exactly one, but occasionally the number can be two or more. Furthermore, it is possible that two mutations that occur in the line of descent from genotype S2 to S3a-x may affect the same genomic position. Thus, L2 does not necessarily represent the Hamming distance between genotypes S2 and S3a-x. Similar observations apply to L3. The external branch length of our structure is simply L3, which ranged in value between 10 and 600. For each environmental model and each setting of L2 and L3, we produced 100 4 taxa phylogenies.

### Generation of Computer Simulation Data

Given the known phylogeny, which includes the exact sequence information of all internal ancestors, we perform several computer simulations that mimic the actual population with varying degrees of accuracy. In all the simulations, we follow the actual phylogeny exactly in terms of number of mutations. That is, if there were exactly 5 mutations from X to Y in the actual phylogeny, there will be exactly 5 mutations from X to Y in the simulated phylogeny. The computer simulations differ only in the mutation location and/or the character substitution models employed.

The control model uses both a uniform mutation location model and a uniform character substitution model. The *location simulation *model uses a uniform character substitution model but emulates exactly the position of all mutations. That is, if the 5 mutations occurred in (for example) positions 3, 15, 23, 47, and 98 of sequence Y in the actual phylogeny, they will also occur in positions 3, 15, 23, 47, and 98 of sequence Y in the simulated phylogeny, though the specific characters involved may differ. Finally, the *substitution simulation *model uses a uniform mutation location model but emulates exactly the substitutions that occur. That is, if the 5 mutations of sequence Y in the actual phylogeny were (a, f), (e, z), (f, t), (s, a), and (z, u) where the first entry of each order pair is the original character in sequence X and the second entry of each order pair is the character after mutation in sequence Y, then we enforce that the same changes occur in the simulated phylogeny, though at potentially different positions.

For each of the 100 Avida trees, we generated a new dataset using each simulation model, resulting in a total of 400 trees (the 100 originals plus 300 simulated trees).

### Generation of PSeq-Gen Data

The PSeq-Gen tool is widely used to simulate the evolution of protein sequences along evolutionary trees. Three substitution models are implemented in PSeq-Gen: JTT, PAM and mtREV. We introduced a uniform substitution model where all the substitutions are equally likely. The only mutation location model implemented in PSeq-gen is the Gamma distribution. A small value for α simulates a large degree of site-specific rate heterogeneity, and as this value increases, the simulated data becomes more rate-homogeneous. We used two α values, 1 and 100. Thus we had a total of 8 unique parameter settings: four substitution models combined with two alpha values. Based on our four taxa model tree, for each parameter setting, we generated 1000 independent replicates. All the simulations use sequence length 100.

## Authors' contributions

DH and ET originated the study. DH performed all the experiments. DH and ET performed all the data analysis. ET, CO, and TS provided feedback on experimental design and analysis in weekly meetings. DH and ET did most of the writing with valuable feedback from CO and TS.
